# Legionella-Induced Hepatitis: A Case Report

**DOI:** 10.7759/cureus.37497

**Published:** 2023-04-12

**Authors:** Devesh Kumar, Naisarg B Vanani, Jared Dobbs, Pinky Jha

**Affiliations:** 1 Medicine, Medical College of Wisconsin, Wauwatosa, USA; 2 Internal Medicine, Medical College of Wisconsin, Wauwatosa, USA

**Keywords:** acute kidney injury, hepatitis, hyponatremia, covid-19, extrapulmonary manifestations, aquatic systems, gram-negative bacillus, pontiac fever, legionnaire’s disease, legionella pneumophila

## Abstract

Legionnaires' disease is caused by a potentially life-threatening infection with the opportunistic Gram-negative bacilli species *Legionella pneumophila*, which is transmitted via inhalation or aspiration of water droplets. Legionnaires’ commonly presents as atypical community-acquired pneumonia with accompanying diarrhea. Although hepatic and renal involvement are relatively uncommon, in this report, we present a case of *Legionella *pneumonia with acute hepatitis.

## Introduction

First discovered in 1976 after an outbreak at the American Legion Convention in Philadelphia, *Legionella pneumophila* is an opportunistic Gram-negative intracellular bacillus that is responsible for causing Legionnaires' disease and Pontiac fever. Legionnaires' disease is commonly seen as atypical community-acquired pneumonia as a result of* Legionella*’s ability to thrive in environments such as aquatic systems, soil, and within biofilms [1]. According to the United States Center for Disease Control (CDC)’s latest surveillance report, the primary mode of transmission of *Legionella* occurs through the aspiration of aerosolized organisms, accounting for approximately 57% of outbreaks of water-based infections and 13% of illnesses from such infections [1,2]. Legionnaires’ most commonly presents as lobar pneumonia and can also present with extrapulmonary manifestations such as mental confusion, dullness, lethargy, myalgias, watery diarrhea, abdominal pain, and bradycardia. Renal and hepatic extrapulmonary manifestations of Legionellosis are rare, and often present with multisystem involvement when they do occur [3]. We present a rare case of a 61-year-old female with a history of anemia and hypertension presenting with *Legionella *pneumonia with acute hepatitis, gastroenteritis, and renal extrapulmonary manifestations.

## Case presentation

**Figure 1:**
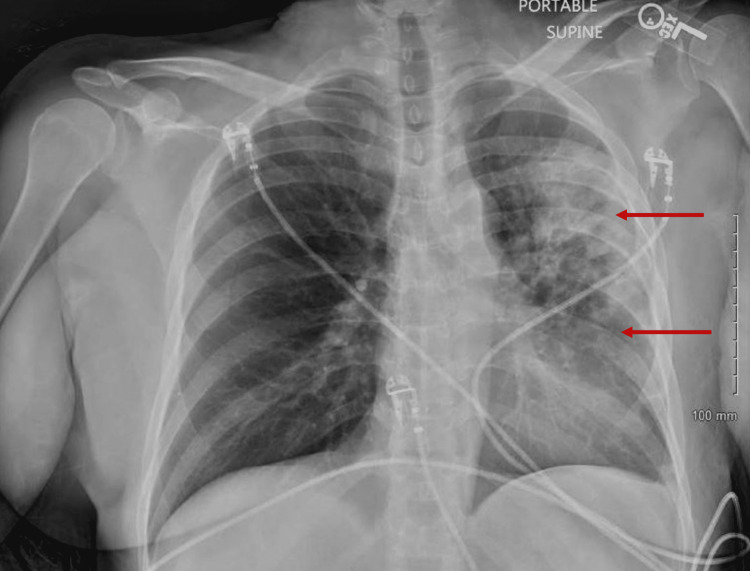
Chest X-ray As noted by the red arrows, the chest x-ray was significant for extensive diffuse left hemithorax consolidation, consistent with pneumonia.

A 61-year-old female with a past medical history of anemia and hypertension presented following several days of loose stools, PO (per oral) intolerance, vomiting, increasing shortness of breath, and a productive cough. Her symptoms began soon after returning from another state with known coronavirus disease 2019 (COVID-19) exposure. Upon presentation, the patient was tachycardic, which improved with intravenous (IV) rehydration. She was otherwise hemodynamically stable, afebrile, and saturating appropriately on room air with rhonchi observed on examination. Initial laboratory results showed an elevated white blood count (WBC) count of 34,000/mL, low sodium of 132 mEq/L, low potassium of 2.6 mmol/L, and elevated creatinine of 1.5 mg/dL. Chest radiograph showed extensive left hemithorax consolidation consistent with pneumonia, after which IV ceftriaxone and oral azithromycin were given in the emergency department (Figure 1).

The CT scan of the Abdomen and Pelvis was negative for any acute findings, with patent hepatic and portal veins, and the absence of any biliary dilatation. An ultrasound (US) of the patient’s right upper quadrant (RUQ) indicated normal echogenicity and was not consistent with hepatomegaly, non-alcoholic steatohepatitis (NASH), portal vein thrombosis, or any other cirrhotic etiology. COVID-19 testing along with blood and urine cultures were negative for the presence of pathogenic species. Given the constellation of gastroenteritis, pneumonia, and hyponatremia, urine *Legionella* antigen testing was performed, which resulted as positive for *Legionella pneumophila* serogroup 1 antigen.

On hospital day (HD) 1, liver function tests (LFTs) were obtained, given the patient’s history of persistent nausea, which showed elevated alanine transaminase (ALT; 406 U/L) and aspartate aminotransferase (AST; 474 U/L). IV ceftriaxone was discontinued after one dose. The patient was rehydrated with IV fluids with electrolyte replacement and a 500 mg/day azithromycin course was continued. The patient’s liver enzymes peaked on HD2 at ALT 619 U/L and AST 687 U/L, which subsequently trended downwards throughout the hospital course with continued antibiotic treatment. Similarly, creatinine levels (1.5 mg/dL on presentation) trended downwards throughout the hospital course following IV rehydration therapy, reaching a nadir of 0.74 mg/dL by HD 5 (Table 1).

**Table 1:** Pertinent Labs Throughout Hospital Course ↑ means the lab value is elevated or above the normal range. ↓ means the lab value is below the normal range. WBC: white blood count, ALT: alanine transaminase, AST: aspartate aminotransferase

Lab Test (Units)​	Hospital Day (HD) 1​	HD 2​	HD 3​	HD 4​	HD 5​
WBC (1,000/mL)​	34​	22.6​	17.8​	12​	N/A​
Sodium (mEq/L)​	132​	136​	137​	139​	140​
Potassium (mmol/L)​	2.6​ ↓	3.1​	3.3​	3.4​	3.5​
Creatinine (mg/dL)​	1.5​ ↑	0.93​	0.92​	0.74​	0.74​
Total Protein (g/dL)​	7.8​	6.1​	6.4​	6.4​	5.7​
Albumin (g/dL)​	3.2​	2.5​	2.6​	2.9​	2.6​
Alkaline Phosphatase (U/L)​	203​	204​	220​	224​	253​
ALT (U/L)​	406​ ↑	619​ ↑	544​ ↑	380​ ↑	343​ ↑
AST (U/L)​	474​ ↑	687​ ↑	414​ ↑	173​ ↑	196​ ↑
Total Bilirubin (mg/dL)​	0.3​	0.2​	0.4​	0.3​	0.4​

No definitive cause was identified when a comprehensive work-up for underlying etiologies was conducted, which included infectious, autoimmune, genetic, metabolic, and thromboembolic conditions. The prothrombin time (PT)/international normalized ratio (INR) results were normal, indicating intact hepatic synthetic function and the hepatitis panel was negative for acute hepatitis infection. The patient had denied any significant alcohol history, which is consistent with the findings on RUQ US along with AST:ALT ratio of <2, lowering suspicion of alcoholic etiology.

By HD 5, the patient’s symptoms had improved with antibiotics and she was discharged with a clear lung examination, resolution of acute kidney injury (AKI), and downward-trending transaminases. Due to the lack of other likely etiologies, along with the consistent improvement of LFTs, creatinine, and resolution of pulmonary symptoms with antibiotic treatment, the diagnosis of *Legionella*-induced acute hepatitis with gastroenteritis was made. The patient was discharged and plans for outpatient monitoring of downward-trending LFTs were made.

## Discussion

Legionnaires' disease is pneumonia caused by the *Legionella pneumophila* bacterium and has been shown to have a mortality rate ranging from 8-12% with higher rates in the nosocomial and elderly population. It is essential to identify rare extrapulmonary manifestations to ensure early treatment [4]. In the post-COVID era, there is an overlap in presentations between COVID-19 and Legionnaires’ which makes the identification of extrapulmonary manifestations even more important. The incubation period for *Legionella* is two to 14 days, commonly presenting as atypical pneumonia with the following symptoms in decreasing prevalence: fever (67-100%), productive cough (41-92%), chills (15-77%), dyspnea (36-56%), headaches and confusion (38-53%), myalgia (20-43%), diarrhea (19-47%), and pleuritic chest pain (14-50%) [1,5,6].

Laboratory abnormalities associated with *Legionella* include hyponatremia, hypophosphatemia, elevated creatine kinase, myoglobinuria, leukocytosis with relative lymphopenia, and elevated inflammatory markers (erythrocyte sedimentation rate [ESR], c-reactive protein [CRP], and ferritin) [1]. In our case, in addition to hyponatremia, leukocytosis, and hypophosphatemia, the patient presented with signs of acute kidney injury with an elevated creatinine level. As this creatinine level resolved with IV fluid rehydration and sufficient oral intake, it was determined that this elevated creatinine was likely due to prerenal azotemia due to her history of recent diarrhea, vomiting, and insufficient oral intake. The potential of this acute kidney injury with Legionnaires’ is an important consideration to make, particularly as it pertains to the administration of renally dosed or nephrotoxic medications. 

Diagnosis of Legionnaires' can be made through the urine antigen test and sputum culture. However, Legionnaires' is often underdiagnosed and underreported [7]. Treatment recommendations include a combination of β-lactam and macrolide antibiotics for three to five days or longer for immunosuppressed patients [1,8]. In our cases, the patient was not immunocompromised, and a five-day macrolide (azithromycin 500 mg/day) regimen was initiated, resulting in the downward trend of LFTs with the resolution of pulmonary and gastrointestinal symptoms of Legionellosis. This confirmed the utility of a macrolide antibiotic for not only isolated Legionnaires’ pneumonia but also for the treatment of multisystem involvement as well. 

Legionnaires’ has been shown to result in hepatic dysfunction, hematuria, and proteinuria at higher rates compared to other causes of pneumonia. In a review of 56 hospitalized Legionnaires’ patients, 25% of their subjects displayed slightly elevated transaminase enzymes, with only two patients having transaminase levels over 100 U/L and one patient experiencing hyponatremia [9,10]. Our patient experienced peak transaminase levels above 600 U/L with hyponatremia and elevated creatinine levels at presentation. Workup for abnormal LFTs ruled out other causes including viral hepatitis and alcoholic hepatitis. This case presents a rare but serious multisystem manifestation of Legionnaires' disease with hepatic dysfunction, hyponatremia, and acute kidney injury. 

While the most common extrapulmonary manifestation is usually in the myocardium, there have also been reports of pyelonephritis and pancreatitis [11,12]. *Legionella*’s seemingly variable extrapulmonary and multisystem involvement can be attributed to the organism's ability to cross the lung epithelium, resulting in bacteremia and multi-system effects [13]. This explanation of *Legionella*’s effects on various organ systems is further corroborated by Watts et al. (1980), who conducted a post-mortem analysis of an immunocompromised patient who succumbed to acute pneumonia caused by Legionnaires’. During this post-mortem analysis via direct immunofluorescence, *Legionella* bacilli were found in the hepatic sinusoids, spleen, lymph nodes, and others [14]. Hence, this suggests a potential model through which *Legionella* manages to have such variable multi-system effects. 

## Conclusions

This case highlights the importance of considering Legionnaires' disease in patients with a combination of pulmonary and gastrointestinal symptoms, as well as monitoring for organ dysfunction, including the liver and kidneys. Early identification and treatment can reduce morbidity and mortality.
